# The Development and Optimisation of a Urinary Volatile Organic Compound Analytical Platform Using Gas Sensor Arrays for the Detection of Colorectal Cancer

**DOI:** 10.3390/s25030599

**Published:** 2025-01-21

**Authors:** Ramesh P. Arasaradnam, Ashwin Krishnamoorthy, Mark A. Hull, Peter Wheatstone, Frank Kvasnik, Krishna C. Persaud

**Affiliations:** 1 Institute of Precision Diagnostics & Translational Medicine, University Hospitals Coventry & Warwickshire, Coventry CV2 2DX, UK; 2 Warwick Medical School, University of Warwick, Coventry CV4 7AL, UK; 3 Leicester Cancer Research Centre, University of Leicester, Leicester LE1 7RH, UK; 4 Leeds Institute of Medical Research, St James’s University Hospital, University of Leeds, Leeds LS9 7TF, UK; 5 Department of Chemical Engineering, The University of Manchester, Manchester M13 9PL, UK

**Keywords:** urinary volatile organic compounds, significant bowel disease, colorectal cancer detection, analytical platform development, solid-phase microextraction, non-invasive screening, radial basis function neural network

## Abstract

The profile of Volatile Organic Compounds (VOCs) may help prioritise at-risk groups for early cancer detection. Urine sampling has been shown to provide good disease accuracy whilst being patient acceptable compared to faecal analysis. Thus, in this study, urine samples were examined using an electronic nose with metal oxide gas sensors and a solid-phase microextraction sampling system. A calibration dataset (derived from a previous study) with CRC-positive patients and healthy controls was used to train a radial basis function neural network. However, a blinded analysis failed to detect CRC accurately, necessitating an enhanced data-processing strategy. This new approach categorised samples by significant bowel diseases, including CRC and high-risk polyps. Retraining the neural network showed an area under the ROC curve of 0.88 for distinguishing CRC versus non-significant bowel disease (without CRC, polyps or inflammation). These findings suggest that, with appropriate training sets, urine VOC analysis could be a rapid, low-cost method for early detection of precancerous colorectal polyps and CRC.

## 1. Introduction

Colorectal cancer (CRC) is the third leading cause of cancer deaths and represents 10% of the global cancer incidence [1]. New cases are expected to increase from 1.15 million (2020) to 1.92 million in 2040 [2]. Screening the population for early signs of CRC reduces cancer mortality, allowing for the removal of precancerous lesions and early-stage CRC [3]. Faecal Immunochemical Tests (FITs) are the most prevalent non-invasive screening method, using an antibody to detect human haemoglobin in a stool sample [4,5,6], followed by colonoscopy if the sample is above a designated set threshold. These sample-positive patients are offered invasive colonic examinations (colonoscopy or CT colonography), and up to 30% will have significant bowel disease (SBD). Significant bowel disease includes colorectal cancer, polyps (precancerous lesions) and inflammatory bowel disease.

A meta-analysis study reports that FITs could detect CRC with a pooled sensitivity of 79% and specificity of 94% [7], leading to a reduction of mortality of 22% in areas where FIT screening programmes were active [8]. In practice, there is a wide variation in sensitivity, and FITs can still miss more than 10% of cancers [9,10]. In screening for cancers, the false-negative rate can vary by 66% depending on the cut-off thresholds used [5,6,11]. Other non-invasive screening methods that might augment the sensitivity of the FIT are desirable, to reduce the risk of missing CRC.

Volatile Organic Compounds (VOCs) originate from several metabolic pathways [12]. Oxidative stress, peroxidation of cell membranes caused by genes or protein alterations in cancer cells may be sources of cancer-related VOCs [13]. They end up in systemic circulation before being excreted in exhaled air, urine and other secretions. Wen et al. [14], in a systematic review of urinary VOCs associated with disease, reported 21 VOCs from eight chemical classes in gastrointestinal cancer patients (gastroesophageal, colorectal and hepato-biliary). These consisted of aromatic compounds, alcohols and ketones. Van Liere et al. [15] more recently analysed 16 studies involving 837 CRC patients, of which 11 carried out chemical identification and 7 chemical fingerprinting, and they reported over 100 VOCs associated with colorectal cancer. These included hydrocarbons, carboxylic acids and aldehydes/ketones, compounds associated with the tricarboxylic acid cycle and amino-acid metabolism. From evidence that VOCs present in urine samples may be indicative of the presence of CRC [16,17], Chandrapalan et al. [18], in a systematic review, concluded that, in an FIT-negative symptomatic population, VOCs can be a good test for ruling out the presence of SBD. While urine sampling is well accepted by patients as well as clinicians because of its simplicity, it is also recognised that urine composition may be affected by a range of factors, including diet, hydration state, time of urination, medication, level of physical activity and others [19,20]. Despite these sample variations, several studies, reviewed by Goertzen et al. (2024) [21], indicate that potential urinary volatile compounds as screening markers for cancer may be detected by several different analytical platforms as well as by dogs [19], and this is corroborated by Hara et al. (2025) [22], who provide a detailed list of volatile compounds found in urine that are associated with different cancers. Previous work has also shown minimal variation of urinary VOCs in those with inflammatory bowel disease [13,23].

In cancers, the genetic changes that result in modified cellular metabolism occur in the initial stages of the disease process, so VOCs may be particularly useful in screening at-risk populations. Traditional laboratory-based methods of analysing volatile chemicals are usually based on gas chromatography–mass spectrometry (GC-MS). This is expensive, time consuming and difficult to use for screening purposes in daily medical practice. As a result, alternative technologies are being investigated. For colorectal cancer, a pilot study using field asymmetric ion mobility spectrometry (FAIMS) to detect volatiles in urine samples showed a promising sensitivity of 88% with a selectivity of 60% [24,25,26]. FAIMS is an effective yet intricate tool that investigates the physical properties rather than the chemical properties of the molecules in question.

Electronic noses (EN) have great potential in this area; they are cheaper, portable instruments based on gas sensor arrays that are relatively non-specific but capable of discriminating complex mixtures of volatile chemicals without necessarily identifying the chemicals present [27]. There is consensus that the changes in the volatilome due to a disease state may apply to several chemical compounds that change in relative concentration, as well as the appearance or disappearance of some chemical species [20,21,28]. The complexity is difficult to disentangle and a holistic method that can discriminate between diseased and non-diseased populations without the necessity of separation and identification of individual chemicals is advantageous. EN have been applied to cancer diagnoses using a variety of sample matrices such as exhaled breath [29], applied to infectious diseases [30,31,32,33] and gastrointestinal diseases [34]. Costantini et al.’s [20] report included an extensive investigation into urinary volatilome profiling by an electronic nose performed using a conducting polymer sensor array in the field of renal carcinoma. Bannaga et al. [35] conducted a series of pilot studies using urine samples where the volatile compounds were preconcentrated by solid-phase microextraction (SPME) and desorption onto a sensor array comprising eight metal oxide gas sensors (SENSAM Ltd., Altrincham, UK) (SPME-EN) and demonstrated that liver, prostate and bladder cancers could be discriminated. It was demonstrated that another electronic nose (PEN3, Airsense Analytics GmbH, Schwerin, Germany) was also able to distinguish CRC from controls, and that the results were comparable to those obtained with gas chromatography–time-of-flight–mass spectrometry (GC-TOF-MS) [16,36].

Here, we present a portion of the results obtained from a multicentre project aimed at reducing colonoscopies in patients without significant bowel disease (the RECEDE study). This was a blinded study, involving measurements of VOCs derived from urine samples using an electronic nose (SENSAM Ltd.), previously described by Bannaga et al. [35]. The initial training set was derived from a previous multicentre observational study (the FAMISHED study). This investigated how the microbiome in the gut changes in relation to different types of inflammatory bowel conditions [37], and urine samples had been collected from patients with a variety of gastrointestinal conditions. We documented the learning experience and how an appropriate strategy was developed as samples were evaluated according to various categories of significant bowel disease. This article focusses on VOC measurements and the data processing associated with this study. The potential advantage of this work is that it can help to increase diagnostic accuracy by a combination of FIT and VOC measurement, both of which are non-invasive and inexpensive compared to endoscopy.

## 2. Materials and Methods

Ethical approval was granted by North West–Liverpool Central Research, UK, as part of the RECEDE study—Ref: 20/NW/0346. The study protocol conformed to the ethical guidelines of the 1975 Declaration of Helsinki, as reflected by the institution’s human research committee. Written informed consent was obtained from all participants prior to their enrollment into the study.

The RECEDE study is a multicentre diagnostic accuracy study involving 17 centres to recruit patients referred for colonic investigations.

The inclusion criteria included the following patients: Patients referred with lower gastrointestinal symptoms through non-urgent pathways as well as those referred urgently with suspicion of CRC in accordance with NICE NG12 criteria [38];

- Patients aged over 16 years;
- Patients able to give informed consent to participate;
- Patients that completed colonic examination;
- Patients that returned both stool and urine samples.

The exclusion criteria excluded the following patients:

- Patients under 16 years of age;
- Pregnant patients.

The interventions carried out were a stool FIT, the analysis of urine VOCs and a comparator colonoscopy (complete examination).

The FAMISHED (Food and Fermentation Using Metagenomics in Health and Disease—09/H1211/38) was a British multicentre observational study that recruited patients with symptoms from a range of gastrointestinal conditions, including colorectal cancer, polyps and inflammatory bowel disease, and healthy individuals, etc.

Those under 16 years of age, or those who were pregnant, and those without final confirmatory diagnoses were excluded.

**Figure 1:**
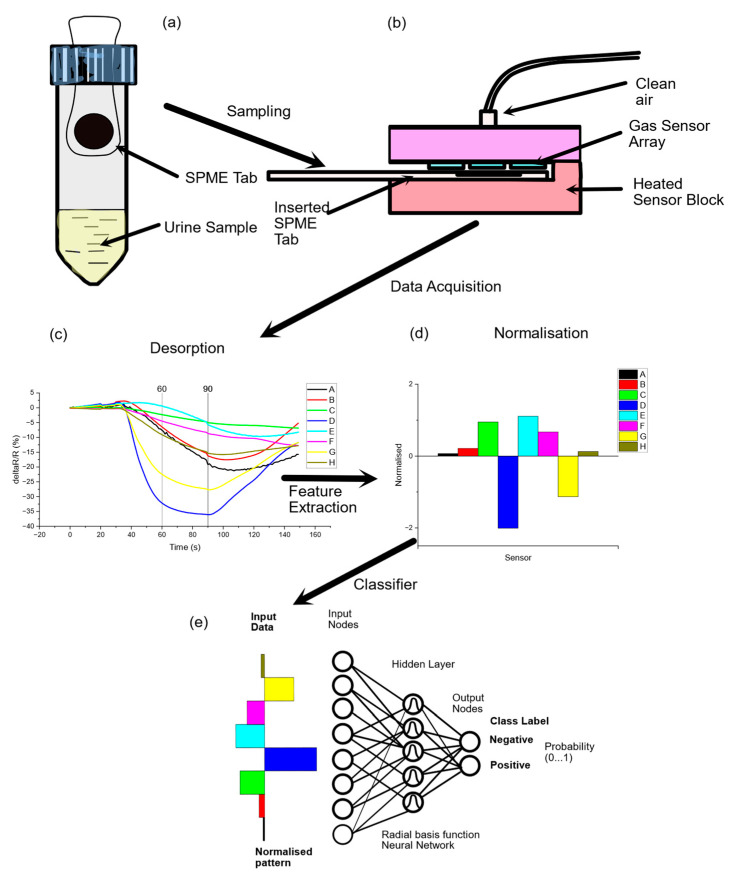
Schematic showing (**a**) sampling of VOCs from urine sample headspace using an SPME tab sampler for adsorption of volatiles, (**b**) desorption of adsorbed volatiles from tab onto a gas sensor array, (**c**) raw data from the array of sensors (each sensor has a different spectrum of response to different volatile chemicals), (**d**) feature extraction to produce a normalised fingerprint that can be added to a database for training a classifier (**e**) comprising a radial basis function neural network.

Urine VOC analysis: Study participants were provided with a universal Sterilin 30 mL pot for the collection of spot urine samples at the time of their clinic visit or for use at home. Samples collected at home had to be frozen with maintenance of the cold chain and be provided more than 4 days (at least) prior to the consumption of bowel-cleansing medication. The timing of collection was recorded prior to storage of the samples at −80 °C. The SENSAM volatile sampling system (SPME-EN, previously described by Bannaga et al. [35]) consists of single-use SPME tabs coated with adsorbent polymers that have differing degrees of selectivity to various volatile chemicals. They can be used for trapping any kind of complex volatile headspace above a liquid matrix. This is analogous to solid-phase microextraction fibres—a technique well established for the preconcentration of headspace samples, normally for gas chromatography–mass spectrometry [39]. The polymer used extracts different kinds of volatile analytes. The quantity of analyte extracted is proportional to its concentration in the sample if equilibrium is reached or after a fixed exposure time. The SENSAM volatile sampling system (SPME-EN) consists of single-use SPME tabs of large surface area coated with adsorbent polymers that have differing degrees of selectivity to various volatile chemicals. They can be used for trapping any kind of complex volatile headspace above a liquid matrix. This is analogous to solid-phase microextraction fibres—a technique well established for preconcentration of headspace samples, normally for gas chromatography–mass spectrometry [39]. The polymer used extracts different kinds of volatile analytes. The quantity of analyte extracted is proportional to its concentration in the sample if equilibrium is reached or after a fixed exposure time. Due to the high diffusion coefficients, gaseous samples have a low extraction time, and control experiments indicated little change in the concentration absorbed after 5 min from urine headspace samples (see Appendix A Figure A2). Duplicate tabs are inserted within the sample headspace for exactly 5 min at ambient temperature (~20 °C) to capture the VOCs (Figure 1a). Thereafter, the SPME tabs are removed and sealed in a plastic receptacle. The SPME tabs, once exposed, can be stored at room temperature for at least a week without substantial loss of trapped volatiles and indefinitely at −80 °C. An SPME tab is inserted into a sensing instrument that comprises an array of eight gas sensors intimately connected to a thermal desorption system that heats it to 120 °C, releasing the volatiles that were previously entrapped on the tab so that they can be measured (Figure 1b). Responses from the array of gas sensors to the desorbed vapours are captured over a period of 150 s, digitised and stored (Figure 1c). The instrument provides a multivariate response that is analysed to produce a fingerprint of the complex mixture of volatiles (Figure 1d) that can be processed using chemometric methods or neural-network-based pattern recognition (Figure 1e) to discriminate between normal and abnormal patient populations and further identify the likelihood of a particular condition that the instrument is trained for. The section of the gas sensor response associated with detecting desorbed volatiles from a single sample (Figure 1c) is isolated and averaged, resulting in n = 5 samples spanning the response profile between 60 and 90 s, as indicated by the cursors. For each patient sample, features extracted from the raw data are processed to generate normalised patterns using z-score normalisation [40] (Figure 1d). Databases of these patterns are then created, serving as the foundation for subsequent data analysis. To visualise the data from all the samples that are processed, the method of principal components analysis (PCA) [41] is used, to provide an unbiased means of observing clusters or patterns when amalgamating samples from a patient population and a control population. Long-term drift in the sensors is corrected by running reference samples whenever a new batch of patient urine samples is processed [42].

To discriminate between different classes of data, a neural network is employed. Several types of neural networks have previously been used for processing data from gas sensor arrays depending on the context (back-propagation feedforward neural networks, radial basis function neural networks, support vector machines, deep learning methods [43,44]). Radial basis function networks (RBFN), a variant of three-layer feedforward neural networks, were used in this study [45]. They contain an input layer, a hidden layer and an output layer where the transfer function in the hidden layer is called a radial basis function (RBF). To each individual pattern in the databases, a class name is assigned. The neural network is trained against a subset of sample patterns and a bootstrap method used for validation and the testing of the prediction accuracy of the neural network against previously unseen samples. The output nodes of the neural network are scaled between 0 and 1 using a SOFTMAX algorithm [46], representing the probability of an input sample pattern belonging to a certain class. This neural network architecture has proven to be robust in this application but does not preclude the use of other approaches as advances in artificial intelligence algorithms are developing rapidly.

A positive likelihood ratio (PLR) is an indicator of how much to increase the probability of having a disease given a positive test result. The ratio is as follows: Probability a person with the condition tests positive (a true positive)/probability a person without the condition tests positive (a false positive). The higher the PLR (>10), the more confidence in the result of a positive test. In this case, the averaged PLR was 80.2 for CRC, providing confidence in the post-test probability (Appendix A Table A1).

A previous observational case cohort study [9,10] using FITs alone for CRC detection and applying a haemoglobin threshold of 3 µg/g faeces revealed a sensitivity of 0.80 [95% confidence interval (CI) 0.66–0.93] and specificity of 0.93 (95%: CI 0.91–0.95). The use of urinary VOCs for the detection of CRC gave a sensitivity of 0.63 (95% CI: 0.46–0.79) and specificity of 0.63 (95% CI: 0.59–0.67). The AUC was 0.67 (95% CI: 0.57–0.77) and the NPV was 0.96 (95% CI: 0.94–0.98). For high-risk polyps and all polyps, using urinary VOCs achieved a sensitivity of 0.93 (95% CI: 0.81–1.0) and 0.91 (95% CI: 0.85–0.97), respectively, with a specificity of 0.16 (95% CI: 0.13–0.20) and 0.15 (95% CI: 0.12–0.19), respectively. The previously published data provided a reference for establishing a sensitivity cut-off threshold for this study’s VOC analysis, suggesting it should be set at 0.63.

To minimise bias, the RECEDE study was a blinded study where no information apart from anonymised patient reference numbers was provided to the partners involved in VOC measurements. The number of patients recruited was 1978, of which 1631 provided urine samples. These were analysed without prior knowledge of the nature of the samples.

Initial analysis of data [9,10] was based on calibration of the instrument on a subset of urine samples taken from a previous research project—the FAMISHED study—that had been retained in storage at −80 °C. Unfortunately, this calibration was limited by differences between study population groups. Here, we document the steps taken to unravel the data acquired from the RECEDE study and summarise the results obtained for VOC measurements of urine samples.

## 3. Results

### 3.1. Reproducibility and Calibration of the Electronic Nose System

Urine samples stored from the previous FAMISHED study were used to calibrate the instrument to differentiate between normal and abnormal patient populations. Table 1 outlines the population demographics within the two studies—FAMISHED (training cohort) and RECEDE (validation cohort). They comprised urine from 53 colorectal-cancer-positive patients and 22 control samples (no disease). For the RECEDE project, samples were processed in batches of approximately 100 over a period of 20 months. Approximately a third of the colorectal-cancer-positive samples used for calibration were measured at the beginning (month 1), month 12 and month 20, at the end of the RECEDE project, while the control calibration samples were measured in month 9. Principal component analysis (PCA) is an efficient technique for visualising multivariate data in two or three dimensions without presupposing any classifications for the samples.

#### 3.1.1. Reproducibility over Time

**Figure 2:**
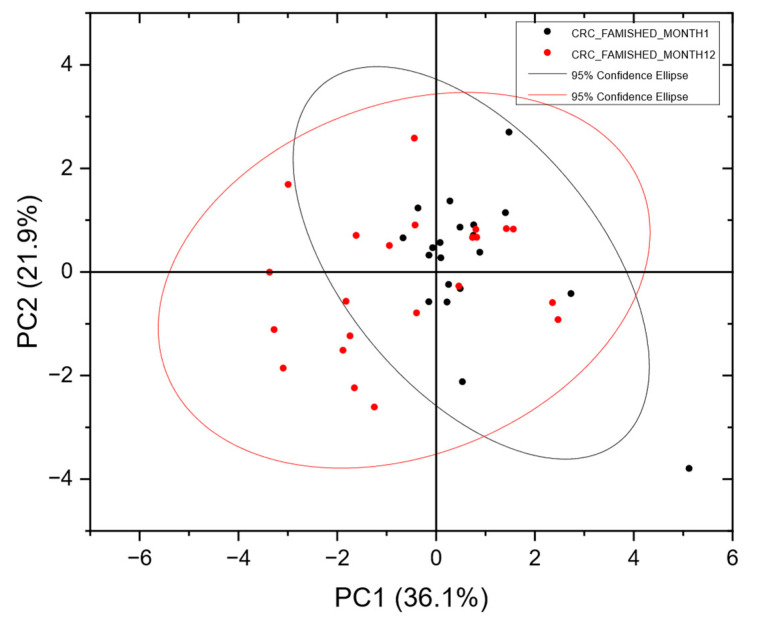
PCA Scores plot of CRC-positive batches from the FAMISHED population measured at month 1 and month 12, showing significant overlap in the two populations, indicating consistency in the CRC samples and repeatable instrument performance. In the Scores plot, each point represents a single urine sample from a subject. The distance between one point and another is dependent on the variance between one VOC pattern and another. Data were analysed without any prior assumptions as to which class they may belong to.

To assess the stability of VOC measurements, Figure 2 presents a PCA Scores plot comparing CRC VOC samples from different patients at month 1 and month 12. The significant overlap between the two populations indicates stable VOC measurements, consistent CRC samples and reliable instrument performance over this period, suggesting a strong potential for developing a robust, trained neural network.

#### 3.1.2. Calibration

**Figure 3:**
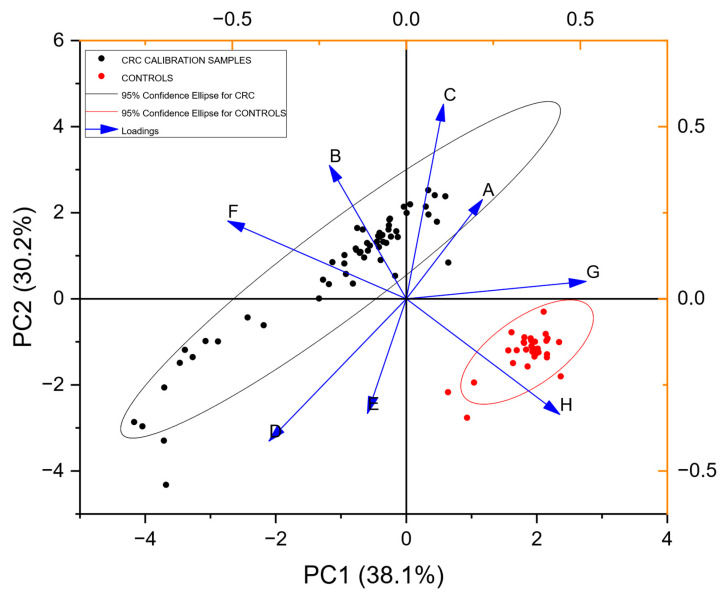
PCA Scores and Loadings plot (training set) for all the calibration samples from populations of CRC-positive and control urine VOCs (Table 1). Principal components axis 1 (PC1) accounts for 38.1% of the information content, while axis 2 (PC2) accounts for 30.2%. The Loadings plot shown with the arrows represents the relative contributions of the individual gas sensors in the array to discrimination between potential classes in the data.

Figure 3 shows a PCA plot resulting from analysis of the calibration samples. Two clusters can be observed visually. The ellipses represent the 95% confidence interval for each cluster. A trained radial basis function neural network correctly classified 100% of each class for the calibration samples.

### 3.2. Blinded Data

**Figure 4:**
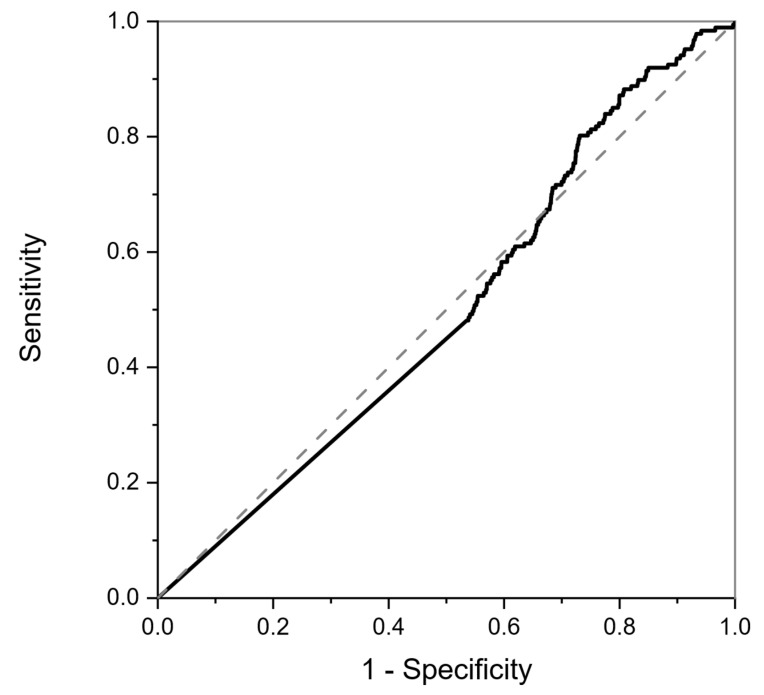
ROC curve for colorectal-cancer-positive samples resulting from classification of RECEDE urine samples in the blinded study. The dotted line is the result of a random classifier, and the solid line is the classifier result of the RECEDE samples. The area under the curve is 0.5, indicating no positive predictive value.

This neural net trained on the calibration data above (Figure 3) was applied in a blinded fashion to the data collected from the RECEDE study (comprising a different unknown population), with each sample being assigned a probability of being positive for disease, e.g., colorectal cancer (CRC), or of being of the non-CRC class, depending on the neural network prediction. Note that, in the RECEDE population, the non-CRC comparator group included those with normal findings as well as those with benign conditions, unlike the FAMISHED cohort where the control group samples were completely normal. The results when applied to the RECEDE population indicated that there was poor positive discrimination between colorectal-cancer-positive samples and non-CRC samples, as illustrated by the ROC curve shown in Figure 4.

### 3.3. Unblinded Data

At this point, the RECEDE study was unblinded and it was then possible to examine the data in detail. Demographic details for the RECEDE population are shown in Table 1 above and disease characteristics in Table 2.

**Figure 5:**
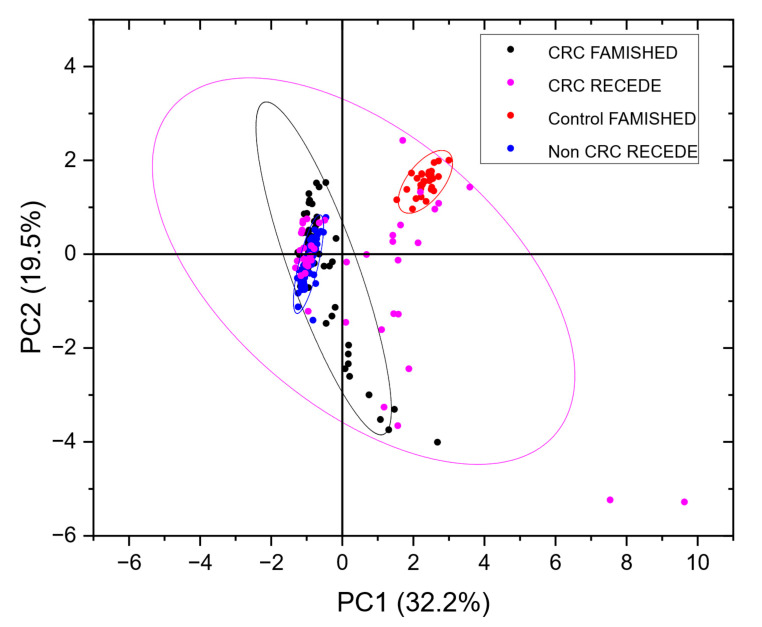
PCA plot of the data shown in Figure 2 (CRC FAMISHED and control FAMISHED) from the FAMISHED study combined with a subset of RECEDE data (CRC RECEDE, non-CRC RECEDE). Full plots of RECEDE data are shown in Appendix A Figure A1.

A principal component analysis was carried out to compare the samples from the FAMISHED study used to train the neural net with the actual samples measured in the RECEDE project. Figure 5 below shows a PCA Scores plot of CRC data from both the RECEDE and FAMISHED studies with their corresponding controls.

Note that, in contrast to the FAMISHED control group, which did not have any bowel disease, for RECEDE, the control group, i.e., the non-CRC group, included those with benign conditions (e.g., diverticular disease, small polyps and microscopic colitis) among normal colonic findings.

In contrast to the tightly grouped clusters seen in the calibration data from the FAMISHED study, the RECEDE CRC-positive data were more dispersed. However, there was still some overlap between the CRC samples from both the FAMISHED and RECEDE cohorts. Conversely, the non-CRC/control RECEDE samples were rather disparate from the control group of FAMISHED samples.

This suggests that the control samples (from the FAMISHED cohort) used to train the neural network were a confounder, as they did not represent the true non-CRC population of the RECEDE study. Notably, the number of control samples available from the FAMISHED study was small (*n* = 22), and there was a gender imbalance (Table 1).

### 3.4. Defining the Control/Comparator Population

To generate a true control population, the RECEDE data were then separated into a population without any benign gastrointestinal conditions termed the NEGATIVE population (as was the case in the FAMISHED control cohort—Table 1). Subsequent re-analysis was undertaken to look at VOC separation for CRC within the RECEDE population.

**Figure 6:**
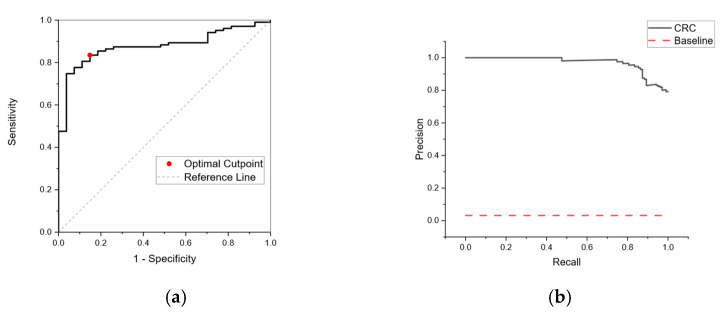
(**a**) Receiver Operating Curve (ROC) for CRC from a neural network retrained on RECEDE data using a NEGATIVE population derived from patient samples with no disease. The neural network is trained against a subset of sample patterns using k-fold validation (5-fold) and a bootstrap method used for training validation. The area under the curve (AUC) was 0.88 (95% confidence limits in the range of 0.78–0.97). (**b**) Precision–recall curve for CRC-positive subjects based on a neural network retrained on RECEDE data. The horizontal dotted line represents a performance of a random classifier. The data indicate a positive predictive value with an AUC of 0.97.

Figure 6 shows an ROC curve resulting from a retrained neural network for RECEDE colorectal cancer data. In contrast to Figure 4 (the network trained on FAMISHED data), the ROC curve shows good positive predictive value with an area under the curve (AUC) of 0.88 and 95% confidence limits in the range of 0.78–0.97.

### 3.5. Resolving Unbalanced Datasets

By testing a sample population, the proportion of individuals with positive conditions can be calculated—the so-called apparent prevalence. From apparent prevalence, the true prevalence in the entire population can be calculated. The prevalence of the CRC population (0.05) is very small compared to the total sample size of those referred for colonoscopy, and hence reliable detection becomes a difficult task. As the sample populations are imbalanced, a negative case for most examples and a positive case for a few examples, an ROC curve in some cases can be misleading. Precision quantifies the number of correct positive predictions made. Recall quantifies the number of correct positive predictions made out of all positive predictions. A precision–recall curve (Figure 6b) was calculated for different probability thresholds. This showed good performance over a random classifier, shown as a horizontal dotted line, confirming the likelihood of positive predictive value for a CRC-positive population.

## 4. Discussion

VOC measurements for detection of various disease conditions are based on differentiation between a diseased population and a normal population. Electronic nose devices are not capable of identifying individual chemical substances as they do not work like mass spectrometers. They can differentiate changes in the composition of complex mixtures of VOCs and this is of use in screening populations—especially if there are no known biomarkers or if they are known but less critical, in which case, the aim would be to identify an ‘at-risk’ population. Several lessons were learnt in this exercise.

The failure of the neural network trained on data from a previous study reflects the importance of understanding the nature of the population to be screened, and the covariates that are associated with the condition to be detected. The neural network was trained on CRC-positive samples and a normal population that had no signs of disease that formed two distinct groups in the FAMISHED study. The RECEDE population, on the other hand, wholly comprised patients referred for further investigation of the colon based on symptoms presented and 72% were on current medication for several different ailments (Table 1). This population (several groups) also comprised subpopulations of patients who had ulcerative colitis, Crohn’s disease and CRC, as well as a proportion with high-risk polyps.

**Figure A1:**
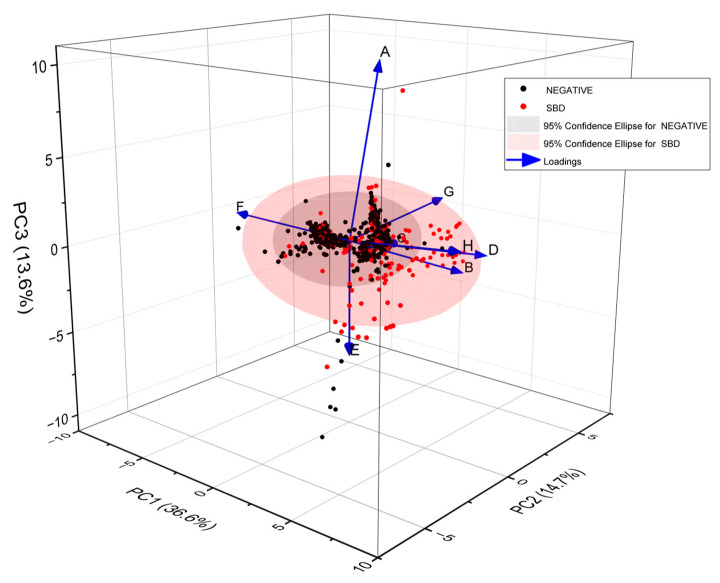
A 3-dimensional PCA plot of all RECEDE samples showing NEGATIVE vs. SBD populations. Each point corresponds to a single urine VOC sample from an individual. The 95% confidence ellipses for NEGATIVE and SBD populations are shown. The arrows show a Loading plot illustrating the contributions of individual sensors in the array.

**Figure A2:**
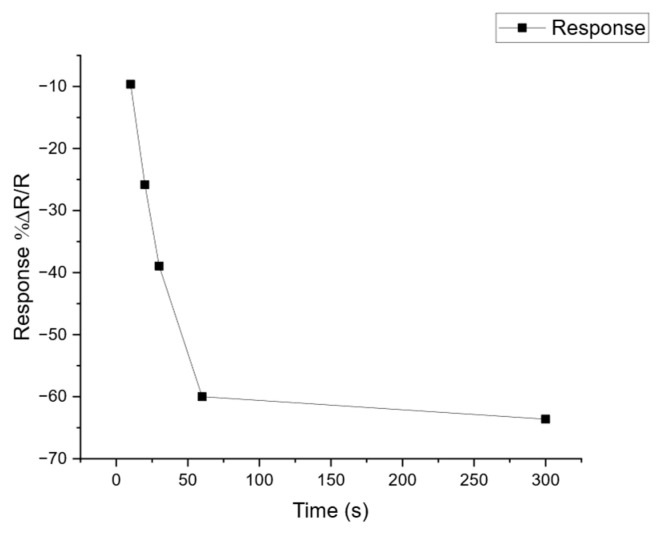
Calibration of SPME tab exposure to volatile analytes. To determine the optimum exposure time for SPME tabs to volatile samples, an experiment was performed to test a range of exposures from 30 s to 300 s against the sensor response. The graph shows the change in amplitude of the sensor response for one of the sensors in the array, expressed as the %ΔR/R, where R is the original resistance of the sensor in air and ΔR the change in resistance observed when exposed to an analyte vapour. The metal oxide sensors show a decrease in resistance when responding to vapour so the curve shows a negative change that tends towards an asymptote after the tab has been exposed for 60 s or more. An exposure time of 300 s was adopted for the RECEDE project.

As can be seen from Figure 5, the control population from the FAMISHED study was disparate from the RECEDE non-CRC/control samples in VOC composition. Whilst the CRC populations of both studies overlapped each other, they also partly overlapped with the RECEDE non-CRC/control population. Hence, it is not surprising that the neural network trained on the FAMISHED samples performed poorly in detection of CRC in the RECEDE samples. Urine composition also reflects covariates such as metabolites excreted because of current medication, other medical conditions such as diabetes and the influence of smoking or alcohol. The PCA plot of Figure A1 (Appendix A), which compares the population in the significant bowel disease (SBD) class (to include colorectal cancer, high-risk polyps (cancer precursors) and inflammatory bowel disease) against the population without any bowel disease (NEGATIVE) confirmed by examination by either colonoscopy or CT colonography, indicates that, despite all of these factors, the diseased population tended to depart from the centroid of the NEGATIVE population and was indeed separable. The diseased population partly overlapped with the negative population, and this was expected to depend on the stage of disease in each patient (detailed disease staging was not available). Notably, however, the variance associated with the diseased population was much higher than the negative population, as shown by the 95% confidence ellipses observed.

The consequence of this study being a blinded study was that there was no a priori information available to guide the data processing of the samples, and the sample population itself was very complex in composition. This highlights a problem associated with the definition of a negative control population. In the RECEDE study, all patients had been referred for further investigation from primary care based on their symptoms. No “healthy” population was available to be compared.

It was reassuring to observe that VOC measurements from different populations of CRC patients measured a year apart overlapped each other (Figure 2). This indicates good stability of the measurement system, and the likelihood that the VOC compositions found in CRC patients have similar profiles.

The retraining of the neural network on RECEDE VOC data inevitably introduced a degree of bias, and, ideally, the neural networks should be tested on a previously unseen population. However, this study indicates the importance of clearly characterising the comparator population and ensuring it is consistent. Despite the perceived bias, there may be some merit in training the neural network using a subset of the population of interest to ensure consistency and help with further optimisation of the neural network (an iterative process). Optimising the neural networks, as well as thresholds for categorising a diseased population, would allow a balance between sensitivity and selectivity that would be acceptable for the screening and longitudinal follow-up of patients. Future studies should factor this in when designing and conducting studies in Volatile Organic Compound analysis.

## Figures and Tables

**Table 1:** Demographics of patients within RECEDE study and FAMISHED study (N/A = not available).

Population	Total Patient Urine Samples	Mean Age (Years)	Male	Female	Mean Height (cm)	Mean Weight (kg)	Mean BMI	Alcohol Users	Smokers	Current Medication
RECEDE (total)	1681	61.0 (sd 13.0)	870 (51.82%)	1101 (65.5%)	168.6 (sd 17.3)	79.6 (sd 18.2)	28.1 (sd 6.2)	966 (57.5%)	1092 (55.4%)	1209 (71.9%)
RECEDE NEGATIVE controls	763	60.4 (sd 12.6)	314	456	168 (sd 10)	80.9 (sd 50.3)	28.5 (sd 14.7)	437 (56.7%)	301 (39.4%)	554 (72%)
FAMISHED controls	22	36.1	8 (36.4%)	14 (63.6%)	N/A	N/A	23.2	12 (54.5%)	4 (18.2%)	N/A
FAMISHED CRC	53	67.3	33 (62.3%)	20 (37.7%)	N/A	N/A	27.2	30 (56.6%)	6 (11.3%)	N/A

**Table 2:** Summary of disease characteristics found in the patient population in the RECEDE study.

Disease Characteristics	% Prevalence
Colorectal cancer (CRC)	4.2
Crohn’s disease (IBD)	1.3
Ulcerative colitis (IBD)	3.4
Polyps (low risk)	52.8
Polyps > 10 mm or high-grade dysplasia < 10 mm, sessile serrated polyps—classed as high-risk polyps/adenomas (HRA)	22.9

## Data Availability

Data reported in this article are available on request to the corresponding author.

## Appendix A

A comparison was made with those in the significant bowel disease (SBD) class (to include colorectal cancer, high-risk adenomas (cancer precursors) and inflammatory bowel disease). The comparator was those without any bowel disease identified (NEGATIVE), confirmed by examination by either colonoscopy or CT colonography. A PCA plot of all the data from the RECEDE study is shown in Figure A1.

This showed that the NEGATIVE population was rather tightly clustered, with a few outliers, while the significant bowel disease population was far more scattered, with a distribution that partially overlapped the NEGATIVE population. The PCA is a limited visual representation of the data and the three principal component axes shown account for about 65% of the information contained in the high-dimension gas sensor dataset. Using a k-fold training–validation (5-fold or 10-fold) approach, with randomisation of data, many neural networks were trained using these data-varying parameters. Methods such as unsupervised cluster analysis were carried out to determine the optimum number of radial basis function centres in the hidden layer of the neural network and optimise the Gaussian kernels of the radial basis function centres. The mean-squared error of prediction was evaluated for each trained network and the five showing the best performance were selected for further evaluation. Results are shown in Table A1 for colorectal cancer (CRC).

**Table A1:** Confusion matrix for RECEDE CRC (*n* = 42) vs. RECEDE NEGATIVE populations (*n* = 777).

Category	False-Negative Rate	False-Positive Rate	True-Positive Rate	True-Negative Rate
	Fraction	Fraction	Fraction	Fraction
CRC	0.03	0.22	0.78	0.97
NEGATIVE	0.22	0.03	0.97	0.78
CRC				
Sensitivity (se)	0.44			
Specificity (sp)	0.99			
Positive predictive value (PPV)	0.78			
Negative predictive value (NPV)	0.97			
Positive likelihood ratio (PLR)	80.24			
Negative likelihood ratio (NLR)	0.56			
